# Comparison of the Effects of Total Laparoscopic Hysterectomy and Total Abdominal Hysterectomy on Sexual Function and Quality of Life

**DOI:** 10.1155/2020/8247207

**Published:** 2020-12-08

**Authors:** Emrah Beyan, Abdurrahman H. İnan, Volkan Emirdar, Adnan Budak, Sadettin O. Tutar, Ahkam G. Kanmaz

**Affiliations:** ^1^Department of Gynecology and Obstetrics, Health Sciences University, Tepecik Education and Research Hospital, İzmir 35180, Turkey; ^2^Department of Gynecology and Obstetrics, Izmir University of Economics, Faculty of Medicine, Izmir 35575, Turkey; ^3^Department of Gynecology and Obstetrics, Giresun University, Faculty of Medicine, Giresun 28200, Turkey

## Abstract

It is known that benign gynecological diseases negatively affect sexual function. For this reason, hysterectomy provides improvement in sexual function as well as symptoms such as bleeding and pain. The effects of abdominal hysterectomy (TAH) and laparoscopic hysterectomy (TLH), which are the two most common types of hysterectomy today, are not clear. In our study, we investigated the effects of TAH and TLH on sexual function and quality of life as well as intraoperative and postoperative results. In 329 TLH and 126 TAH patients, we compared both and between themselves preoperatively and postoperatively by using the standardized and validated female sexual function index (FSFI) and European quality of life five-dimension scale (EQ-5D). In conclusion, we found that both types of hysterectomy were effective in improving sexual function, and we concluded that improvement in the laparoscopy group was statistically higher. Patients who require hysterectomy for benign gynecological reasons should be informed that TLH has a more positive effect on sexual function as well as other advantages, and if the patients' main complaint is sexual dysfunction, TLH should be preferred compared to TAH.

## 1. Introduction

Hysterectomy is one of the gynecological operations performed due to benign indications such as uterine leiomyoma, dysfunctional uterine bleeding, endometrial hyperplasia, dysmenorrhea, dyspareunia, and severe endometriosis refractory to medical or conservative surgical management or in cases of rejection by the patient [1]. Many symptoms that emerge prior to hysterectomy such as heavy and prolonged menstrual bleeding, chronic pelvic pain, dyspareunia, and dysmenorrhea negatively affect the quality of life and sexual function [2]. It can be expected that a hysterectomy to be made to relieve these symptoms can also show positive effects on patients' quality of life and sexual function [3].

TAH (TAH) is an accepted, effective, and easily performed procedure by any gynecologist and is a more invasive technique than TLH (TLH). Today, the rate of TLH among hysterectomies is increasing [4]. Blood loss, infection rate, and postoperative pain are less common in TLH than TAH, and return to daily routine is quicker [5]. Although TLH is considered as a superior method, complications may occur, like urinary system injuries in particular, which are related to the technique and the clinical experience of the physician performing the operation [6–8].

This study is aimed at investigating the effect of surgical technique on women's life by evaluating the short- and long-term outcomes and sexual functions of patients who underwent TLH and TAH due to benign indications.

## 2. Materials and Methods

This is an observational cohort study conducted on patients undergone hysterectomy between May 2014 and May 2018 in a tertiary training and research hospital. For the study, 793 patients who underwent hysterectomy due to benign indications were screened. A total of 455 patients whose data were fully available and accessible with complete data and contact details for communication were included in the study. Of the 455 patients in the study, 329 underwent TAH, and 126 underwent TLH; all operations were done by experienced surgeons who have at least 4 years of experience in the fields. The patients who refused to fill out sexual function and quality of life questionnaires or who refused to answer some of the questions were excluded from the study, and this stage of the study was completed with 259 patients in the TAH group and 96 patients in the TLH group.

The study included premenopausal, sexually active patients who underwent hysterectomy due to benign gynecological indications and who did not undergo oophorectomy. Patients whose operation were started laparoscopically and converted to laparotomy due to intraoperative complications; who were operated for indications such as pelvic organ prolapsus, abscess, bilateral ovarian cysts, and premalignant lesions requiring surgery in addition to hysterectomy; and who are postmenopausal or sexually inactive were excluded from the study. The approval for the study was obtained from the ethics committee of our hospital.

The data of the patients included in the study were retrospectively reviewed from the hospital information system, patient files, examination forms, and operative notes. The patients who were eligible for the study were contacted by telephone and invited to the hospital. After the detailed verbal and written consent of patients, in addition to the follow-up examination, FSFI and EQ-5D questionnaires were administered for preoperative and postoperative periods separately. For patients who cannot visit the hospital, the questionnaire was studiously administered preoperatively and postoperatively by the health care staff to those who accepted. In our clinic, preoperative gynecological examination and ultrasonographic evaluation are performed routinely for all patients scheduled for operation, and preoperative anesthesia consultation is requested. At the same time, in accordance with our clinical policy, each patient is informed about alternative medical treatment and the advantages and disadvantages of the planned surgical technique, and verbal and written informed consent is obtained from the patients. All patients included in the study underwent the same clinical approach as described above.

In our clinic, prophylactic antibiotics are administered to all patients undergoing hysterectomy at least half an hour before the operation, and low molecular weight heparin treatment is given for postoperative thromboembolism prophylaxis. While a drain is placed in patients undergoing TAH if the surgeon requires, a drain is placed in the left or right lower drainage area in all patients undergoing TLH and is removed within 18-24 hours if no complication occurs. Operative times were recorded from the operative notes and standardly determined from the onset of skin incision until the completion of closure in abdominal hysterectomies and from incision of initial trocar entry until the closure of the vaginal cuff in laparoscopic hysterectomies. All abdominal hysterectomies were performed according to the Richardson method, while laparoscopic hysterectomies were performed using a RUMI II System (CooperSurgical, CT, USA) uterine manipulator, and one or two 10 mm and two or three 5 mm trocars were placed. For sealing and cutting large vessels and ligaments, a 5 mm-37 cm LigaSure (Covidien, CO, USA) was used, and a monopolar hook was used for colpotomy. In all patients included in the study, the vaginal cuff was closed up via intracorporeal route.

FSFI is a sexual function assessment tool validated for Turkish, consisting of 2 questions for desire, 4 questions for arousal and lubrication, and 3 questions for orgasm, satisfaction, and pain, a total of 6 subsections and 19 questions [9]. After all, questions are answered, a score ranging from 2 to 36 is calculated, and a total score below 26.55 is considered as sexual dysfunction [10]. EQ-5D is an approved tool for measuring health-related quality of life [11]. It is a validated, self-administered questionnaire consisting of two parts; in the first part, 6 dimensions including mobility, personal care, general activity, social activity, pain, and anxiety/depression are evaluated in three levels of severity which are no problem, mild or sometimes problem, and serious problem. EQ-5D index score ranges from “1 point” indicating full quality of life to “-0.207” point indicating the worst quality of life. The second part consists of a scale, called the vertical visual analog scale (VAS), in which the patient is asked to place a mark on the scale to indicate her current health status by giving scores from 0 to –100.

The general demographic data, reasons of surgery, comorbidities, intraoperative, and postoperative complications, as well as preoperative and postoperative FSFI and EQ-5D questionnaires of the patients included in the study were compared between the two groups according to the operation technique.

### 2.1. Statistical Analysis

All data are presented as mean ± standard deviations (SDs) for continuous variables and median (ranges) and number of cases (*n*) and percentage of occurrence (%) for categorical variables. Normality of the data distribution was evaluated using the Kolmogorov-Smirnov test. All hypothesis tests were carried out using a *p* value of 0.05 for statistical significance. The differences in the distribution of categorical variables between groups were analysed by the chi-squared test and Fisher's exact test. All statistical analyses were performed using the Statistical Package for the Social Sciences (SPSS, version 22.0 for Windows; SPSS, Inc., Chicago, Illinois, USA).

## 3. Results

The study included 329 TAH and 126 TLH patients who were operated due to benign indications and done by experienced surgeons. The age, body mass index (BMI), uterine dimensions, comorbidities, medical histories, and hysterectomy indications are summarized in Table 1 under the heading of demographic and clinical characteristics. There was no statistically significant difference between the demographic data of both groups. At the same time, the most common indication for hysterectomy was uterine myoma in both groups.

The operative and postoperative variables are summarized in Table 2. Estimated blood loss, additional analgesic requirement, duration of drainage, and gas output time were statistically significantly less in the TLH group (*p* < 0.001). Only the mean operative time was shorter in the TAH group, but no statistically significant difference was reported (*p* = 0.112).

Intraoperative and postoperative complications are summarized in Table 3. While intraoperative complications were detected in 13 (3.9%) patients in the TLH group and 7 (5.5%) patients in the TAH group, there was no statistically significant difference between the groups (*p* = 0.455). Postoperative complications were observed in 10 (3.0%) patients in the TLH group and 12 (9.5%) patients in the TAH group, and the results were statistically significant (*p* = 0.004).

Since 70 patients in the TLH group and 30 patients in the TAH group refused to answer our sexual function and quality of life questions, this subgroup analysis of the study was performed with 259 TLH and 96 TAH patients. While there was no statistically significant difference between the two groups regarding preoperative mean FSFI score (*p* = 0.203), the mean postoperative FSFI score of the TLH group was significantly higher (*p* < 0.001). Although there was an increase in the postoperative FSFI scores in both groups, the increase in the TAH group was not statistically significant (*p* = 0.057). The preoperative EQ-5D score was statistically lower in the TAH group than in the TLH group (*p* < 0.001). There was a statistically significant increase in the EQ-5D score in both TLH and TAH groups (*p* < 0.001 and *p* < 0.001, respectively). The increase in the TAH group was statistically significantly higher (*p* = 0.020). Although there was a statistically significant increase in the VAS scores of both groups (*p* < 0.001, *p* < 0.001), there was no statistically significant difference between the groups regarding mean preoperative and postoperative VAS scores and score rises (*p* = 0.388, *p* = 0.745, and *p* = 0.599, respectively) (Table 4).

The mean desire and arousal scores of the TLH group were statistically higher both in postoperative period and in the difference between preoperative and postoperative periods (*p* ≤ 0.001/*p* = 0.002 and *p* = 0.001/*p* = 0.002, respectively). There was no statistically significant difference between the groups in terms of preoperative and postoperative mean regarding lubrication, satisfaction and pain scores, and score rise. Postoperative orgasm score was statistically significantly higher in the TLH group (*p* = 0.002), while the increase in orgasm score was insignificant (*p* = 0.091) (Table 5).

## 4. Discussion

The advantages of laparoscopic hysterectomy, such as less blood loss, less postoperative pain, and shortening of return to daily routine compared to abdominal hysterectomy, which was first described by Reich et al. [12] in 1989 have been demonstrated by randomized studies in the literature [5, 13, 14]. However, although urinary tract injury has been described as a specific complication of laparoscopic hysterectomy in the literature, there are also publications reporting that the risk of urinary tract injury is not much in laparoscopic technique compared to abdominal technique [6, 13, 15–18]. In the study by Janssen et al. [19] investigating the causes of urinary tract injuries in TLH, it was shown that inadequate training and uterine artery sealing without the use of manipulators increased the risk. We think that the reason for no difference between the groups in terms of urinary tract injury rate is the experience of surgeons and the use of uterine manipulator with a cervical cap.

For many years, the idea that a healthy uterus and cervix have an essential role in sexual function has been adopted [20]. However, in the last decade, there are different publications reporting the positive results of hysterectomy performed due to benign gynecological reasons on sexual functions [21–23]. Symptoms such as chronic pelvic pain and abnormal uterine bleeding, which are indications for hysterectomy, adversely affect sexual function. Therefore, the elimination of symptoms in the postoperative period and psychologically more comfortable feelings of the patient will contribute to the improvement of sexual function [2, 24, 25]. In our study, no statistical difference was found between the preoperative FSFI scores. According to some studies in the literature, we think that the reason that the preoperative FSFI scores of both groups were lower was due to ethnic and cultural reasons [26]. Although FSFI scores increased in both groups in our study, the increase in the TAH group was not statistically significant, whereas the mean postoperative FSFI score was statistically significantly higher in the TLH group. In laparoscopic surgery, the improvement in sexual function of patients in the postoperative period can be explained by less intra-abdominal adhesions, less shortening in the vaginal length, and higher body perception. The study by He et al. [26] supports our study findings. In our study, only the mean scores of desire, arousal, and orgasm parameters, which mainly constitute the psychological components of sexual function, were significantly higher in the TLH group in the postoperative period. We believe that the improvements in these parameters are associated with shorter time to return to daily routine and the nonimpaired body perception in patients undergoing laparoscopic surgery [27].

The mean EQ-5D and VAS scores of both groups included in the study were significantly higher in the postoperative period compared to the preoperative period. The randomized controlled study by Kluivers et al. [28], which has a similar design with our study, found similar results; however, Xiao et al. [29] found no statistically significant difference in the change of life quality between laparoscopic and abdominal groups of early stage cervical cancer patients that are treated with radical hysterectomy alone.

Nonaddition of oophorectomy to hysterectomy in the patients included in our study, premenopausal and sexually active patients, and the use of standardized and validated tools such as FSFI and EQ-5D are the strengths of our study. However, the lack of optimal randomization due to the retrospective presence of patients in our study could be the weakness of the study.

## 5. Conclusions

Hysterectomy operation performed due to appropriate indications is expected to have a positive effect on impaired quality of life and sexual function. According to the literature, TLH is superior to TAH in terms of returning to daily routine, especially in the short- and long-term postoperative and sexual function, but it has been reported that vaginal hysterectomy is superior to both methods in patients who are suitable. In our study, we found that both TLH and TAH were effective in improving sexual function, and we concluded that improvement in the laparoscopy group was statistically superior. However, there was no statistical difference between the groups in the increase of the quality of life. Patients who require hysterectomy for benign gynecological reasons should be informed that laparoscopic hysterectomy has a more positive effect on sexual function as well as other advantages, and if the patient's main complaint is sexual dysfunction, laparoscopic hysterectomy should be preferred compared to abdominal hysterectomy, when vaginal hysterectomy is not indicated. We believe that prospective multicentred randomized controlled trials to be conducted in this respect will provide more accurate results.

## Figures and Tables

**Table 1:** Baseline characteristics of patients and indication for surgery.

Characteristics	TLH (*n* = 329)	TAH (*n* = 126)	*p* value
Age (y)	46.4 ± 5.9	46.9 ± 6.1	0.423
BMI (kg/m^2^)	23.7 ± 3.1	23.4 ± 2.8	0.344
Uterine volume			
Normal size	83 (25.2%)	33 (26.2%)	0.833
6-8 weeks gestational size	59 (17.9%)	21 (16.6%)	0.751
8-10 weeks gestational size	98 (29.8%)	34 (27.0%)	0.555
10-12 weeks gestational size	44 (13.4%)	18 (14.3%)	0.800
12-14 weeks gestational size	26 (7.9%)	11 (8.7%)	0.773
>14 weeks gestational size	19 (5.8%)	9 (7.2%)	0.587
Prior cesarean delivery	63 (19.1%)	26 (20.6%)	0.721
Previously undergone abdominal surgery	37 (11.2%)	16 (12.7%)	0.666
Medical comorbidities	60 (18.2%)	22 (17.5%)	0.847
Indication			
Myoma uteri	215 (65.3%)	83 (65.9%)	0.916
DUB	61 (18.5%)	22 (17.5%)	0.789
Endometrial hyperplasia	23 (7.0%)	9 (7.1%)	0.955
Adenomyosis	15 (4.6%)	6 (4.7%)	0.927
CIN3	15 (4.6%)	6 (4.7%)	0.927

TLH: total laparoscopic hysterectomy; TAH: total abdominal hysterectomy; BMI: body mass index; DUB: dysfunctional uterine bleeding; CIN3: cervical intraepithelial neoplasia grade III.

**Table 2:** Operative and postoperative variables of TLH group vs. TAH group.

Variables	TLH (*n* = 329)	TAH (*n* = 126)	*p* value
OT (min)	100.4 ± 28.1	95.6 ± 30.4	0.112
EBL (mL)	160.5 ± 132.8	285.9 ± 156.2	<0.001
The number of patients that required analgesics (*n*)	55	54	<0.001
Days with indwelling catheter (day)	0.8 ± 0.4	1.5 ± 0.6	<0.001
Passing flatus (hour)	20.8 ± 12.8	28.6 ± 16.5	<0.001

TLH: total laparoscopic hysterectomy; TAH: total abdominal hysterectomy; OT: operative time; EBL: estimated blood loss.

**Table 3:** Intraoperative and postoperative complications of TLH group vs. TAH group.

Complications	TLH (*n*, %) (*n* = 329)	TAH (*n*, %) (*n* = 126)	*p* value
Total types of intraoperative complications	13 (3.9%)	7 (5.5%)	0.455
Bladder or ureter injuries	7 (2.1%)	3 (2.4%)	
Bowel injuries	2 (0.6%)	2 (1.6%)	
Vascular injuries	2 (0.6%)	2 (1.6%)	
Subcutaneous emphysema	2 (0.6%)	–	
Total types of postoperative complications	10 (3.0%)	12 (9.5%)	0.004
Febrile event	3 (0.9%)	3 (2.4%)	
Wound infection	1 (0.3%)	2 (1.6%)	
Bleeding or hematoma	1 (0.3%)	2 (1.6%)	
Urinary retention	2 (0.6%)	1 (0.7%)	
Ileus	1 (0.3%)	2 (1.6%)	
UTI	2 (0.6%)	2 (1.6%)	

TLH: total laparoscopic hysterectomy; TAH: total abdominal hysterectomy; UTI: urinary tract infection.

**Table 4:** Outcomes of FSFI, EQ-5D (TTO), and VAS.

Characteristics	TLH (*n* = 259) Mean ± SD	TAH (*n* = 96) Mean ± SD	*p* value
FSFI			
Preoperative	18.87 ± 6.63	17.89 ± 5.87	0.203
Postoperative	21.65 ± 5.42	19.35 ± 4.62	<0.001
Difference	2.78 ± 5.65	1.46 ± 4.97	0.044
Difference *p* value	<0.001	0.057	
EQ-5D (TTO)			
Preoperative	0.79 ± 0.19	0.71 ± 0.21	<0.001
Postoperative	0.86 ± 0.21	0.85 ± 0.16	0.672
Difference	0.07 ± 0.19	0.14 ± 0.18	0.002
Difference *p* value	<0.001	<0.001	
VAS			
Preoperative	63.22 ± 25.93	60.61 ± 23.37	0.388
Postoperative	81.27 ± 17.64	80.59 ± 17.12	0.745
Difference	18.05 ± 31.71	19.98 ± 27.61	0.599
Difference *p* value	<0.001	<0.001	

TLH: total laparoscopic hysterectomy; TAH: total abdominal hysterectomy; FSFI: Female Sexual Functioning Index; EQ-5D: European quality of life five-dimension scale; VAS: vertical visual analog scale.

**Table 5:** Outcomes of FSFI subcategories.

FSFI subcategories	TLH (*n* = 259) Mean ± SD	TAH (*n* = 96) Mean ± SD	*p* value
Desire			
Preoperative	2.87 ± 1.26	2.70 ± 1.11	0.245
Postoperative	3.54 ± 1.25	2.81 ± 1.36	<0.001
Difference	0.67 ± 1.58	0.11 ± 1.28	0.002
Difference *p* value	<0.001	0.540	
Arousal			
Preoperative	3.16 ± 1.77	2.93 ± 1.79	0.279
Postoperative	3.86 ± 1.73	3.10 ± 1.88	<0.001
Difference	0.70 ± 2.04	0.17 ± 1.61	0.022
Difference *p* value	<0.001	0.522	
Lubrication			
Preoperative	2.49 ± 1.25	2.27 ± 1.19	0.137
Postoperative	2.56 ± 1.06	2.37 ± 1.08	0.136
Difference	0.07 ± 1.06	0.10 ± 0.98	0.809
Difference *p* value	0.492	0.543	
Orgasm			
Preoperative	2.82 ± 1.64	2.59 ± 1.75	0.250
Postoperative	3.19 ± 1.46	2.64 ± 1.52	0.002
Difference	0.37 ± 1.64	0.05 ± 1.40	0.091
Difference *p* value	0.007	0.833	
Satisfaction			
Preoperative	3.61 ± 1.89	3.54 ± 1.99	0.760
Postoperative	4.12 ± 1.89	3.74 ± 1.75	0.870
Difference	0.51 ± 2.01	0.20 ± 1.52	0.171
Difference *p* value	0.002	0.461	
Pain			
Preoperative	2.53 ± 1.12	2.32 ± 1.21	0.126
Postoperative	4.11 ± 2.21	3.85 ± 1.01	0.268
Difference	1.58 ± 1.76	1.53 ± 1.51	0.805
Difference *p* value	<0.001	<0.001	

TLH: total laparoscopic hysterectomy; TAH: total abdominal hysterectomy; FSFI: Female Sexual Functioning Index.

## Data Availability

The data of our research are of course open to other researchers. However, if there is such a request, we expect it to be requested from us. In this case, the author information to be contacted is as follows. We guarantee that the authors will not encounter any difficulties.
